# Sickle cell trait knowledge and health literacy in caregivers who receive in‐person sickle cell trait education

**DOI:** 10.1002/mgg3.327

**Published:** 2017-08-23

**Authors:** Susan Creary, Ismahan Adan, Joseph Stanek, Sarah H. O'Brien, Deena J. Chisolm, Tanica Jeffries, Kristin Zajo, Elizabeth Varga

**Affiliations:** ^1^ Nationwide Children's Hospital Columbus Ohio

**Keywords:** Health literacy and knowledge, sickle cell disease, sickle cell trait education

## Abstract

**Background:**

Despite universal screening that detects sickle cell trait (SCT) in infancy, only 16% of Americans with SCT know their status. To increase SCT status awareness, effective education for patients and their families is needed. The objective of this study was to assess caregivers’ SCT knowledge before and after an in‐person SCT education session.

**Methods:**

A trained educator provides in‐person SCT education to caregivers of referred infants with SCT at Nationwide Children's Hospital. From August 2015 to July 2016, primarily English‐speaking caregivers of infants with hemoglobin S‐trait were recruited and completed a health literacy assessment and a SCT knowledge assessment (SCTKA) before and after receiving education. Caregivers repeated the SCTKA again after ≥6 months, if they could be contacted.

**Results:**

Thirty‐eight (38.1%) percent of 113 caregivers had high SCTKA scores (≥75%) before education but 90.3% achieved high scores after education. Caregivers with low SCTKA scores after education had significantly lower health literacy (*P* = 0.029) and baseline SCTKA scores (*P* = 0.003) compared to those with higher scores after education. At ≥6 months, caregivers’ scores were significantly higher (*P* = 0.014) than baseline, but only 73.3% scored ≥75%.

**Conclusion:**

Our results suggest that caregivers’ baseline SCT knowledge is low, improves with in‐person education but may decline with time. Caregivers who do not achieve high SCT knowledge after education had lower health literacy and baseline knowledge. Future studies should determine if adapting in‐person education to caregivers’ health literacy and knowledge levels results in high and sustained SCT knowledge among all caregivers and more individuals who know their SCT status.

## Introduction

Sickle cell disease (SCD) is an autosomal recessive, chronic blood disorder that affects approximately 100,000 individuals in the U.S. and leads to substantial morbidity and premature mortality (Centers for Disease Control Data and Statistics, Atlanta, GA; Platt et al. 1994). Nearly 3 million people or 8% of African Americans have sickle cell trait (SCT) (Centers for Disease Control Data and Prevention, Atlanta, GA). While individuals with SCT typically remain asymptomatic and do not suffer significant morbidity, these individuals are at risk of having a child with SCT or SCD (Centers for Disease Control and Prevention; Liem et al. 2017).

In the U.S., hemoglobinopathy newborn screening identifies infants with SCD and SCT and has been widely implemented since the 1980s and 1990s (AAP Newborn Screening Task Force, 2000). This screening resulted in rapid identification and a significant reduction in the mortality of children with SCD (Morbidity and Mortality Weekly Report, 1998). While children with SCT are identified through screening, only 16% of individuals with SCT who are of childbearing age know their status (Treadwell et al. 2006), and the lack of widespread and coordinated SCT testing outside of the newborn period may contribute to this knowledge gap. Most newborn screening programs, including Ohio's, do not offer or provide resources for SCT testing for parents of children identified as having SCT (Ohio Department of Health, 2014) and the Centers for Disease Control SCT Toolkit recommends that these individuals seek and obtain SCT testing from their physician or a local SCD non‐for‐profit (Centers for Disease Control and Prevention: Sickle Cell Trait Toolkit). Genetic counseling has been shown to increase disease knowledge and foster communication within at‐risk family members (Bernhardt et al. 2000), yet physician surveys suggest that 20% of parents of children with SCT do not receive any counseling (Moseley et al. 2013).

Newborn screening programs in the U.S. are designed and managed by each state and are primarily funded by collecting fees for newborn screening from Medicaid and private insurance (Johnson et al. 2006). This leads to significant variation as to who receives SCT testing results, how children and their families are notified of these results, and how SCT education is provided. For example, Florida and Louisiana provide infants’ newborn screen results only to primary care providers and then rely on the primary care providers to provide SCT education to patients and families (Kavanagh et al. 2008). In contrast, Ohio provides in‐person education to caregivers of infants with SCT to increase SCT awareness, make caregivers knowledgeable about SCT so that they can inform their children about SCT when they are older, and make more parents aware that they are at risk for SCT (Ohio Department of Health: Sickle Cell Services 2014). It is unknown, however, if this in‐person education is effective or if caregivers’ SCT knowledge is sustained after receiving this education. Understanding caregivers’ baseline SCT knowledge, the impact of in‐person education on caregivers’ knowledge, and the factors that are associated with achieving high SCT knowledge will better inform how to effectively deliver SCT education. Therefore, the primary aim of this study was to assess caregivers’ SCT knowledge before and after an in‐person SCT education session and to identify barriers to achieving high SCT knowledge. We also aimed to determine if SCT knowledge was sustained, if the education provided reassurance about SCT, and if caregivers were satisfied with their education and intended to inform their children and others about SCT.

## Materials and Methods

### Ethical compliance

This study was approved by the Nationwide Children's Hospital (NCH) Institutional Review Board.

### NCH SCT center

The Ohio Department of Health has an established referral‐based SCT education program for caregivers of infants with SCT who are identified by the newborn screening program. Nationwide Children's Hospital (NCH) serves as the referral center for infants born with SCT in 33 central Ohio counties. The NCH team includes a pediatric hematologist, a program coordinator, an administrative assistant, and a trained SCT educator. The educator and administrative assistant provide case management to assist with referrals and appointments. This program has resources to educate families of all infants born with SCT and typically, 500 of the 800 infants born in this region annually have at least one caregiver attend the education.

### SCT education at NCH

The SCT educator at NCH received hemoglobinopathy education at a 2‐day Hemoglobinopathies Counselor Training Course, observed the licensed genetic counselors in the hematology/oncology clinic at NCH, and had 3 years of SCT education experience prior to the study period. Multiple caregivers of each infant with SCT are able to attend this in‐person education appointment. The education utilizes visual aids and begins with a review of genes and traits, hemoglobin and its function in the body, SCD, and what causes SCD. After this, the educator discusses the common abnormal hemoglobin traits that can lead to SCD (hemoglobin S, hemoglobin C, and *β* thalassemia traits). Caregivers are informed that individuals with SCT are typically asymptomatic and that SCT requires specialized testing to be detected. Finally, the educator reviews different Punnett square diagrams to illustrate the risks of having a child with SCT or SCD depending on SCT status of each parent. Translator services (an interpreter or language line) are available to caregivers during their session, but the educator provides verbal communication in English and the visual aids are only available in English. Each session lasts approximately 20 min but can be extended if caregivers have additional questions or if the educator perceives that caregivers have not understood the material. A SCT pamphlet and the educator's contact information are provided at the end of the session.

### Study design, participants, and recruitment

This was a cross‐sectional, prospective study of caregivers (biological parents or legal guardians) of infants with SCT identified by newborn screening who received in‐person SCT education at NCH. Given the nuances in the education that is provided about Hemoglobin S‐trait compared to the other types of SCT (e.g., Hemoglobin C‐trait), only caregivers of infants with Hemoglobin S‐trait were approached. From August 25, 2015 to July 12, 2016, the NCH educator consecutively approached caregivers of infants with Hemoglobin S‐trait if they presented for education and were at least 18 years old. To avoid including caregivers with any language barriers that could impact the ability to understand the SCT education or interpret the surveys, caregivers who did not report that they were primarily English speaking were excluded. Also, caregivers who previously attended a newborn screening session for any abnormal hemoglobinopathy trait or personally had SCD were excluded, since these caregivers had the potential to have a different baseline SCT knowledge compared to caregivers who had not previously received education. If multiple eligible caregivers for a single child with SCT presented for the education, each was allowed to participate but was required to complete all of the surveys independently. Biologic and step‐parents were considered to be high‐risk caregivers, since these individuals had either a 50% chance of also having SCT themselves or a partner with a 50% chance of having SCT.

### Study procedures

After enrollment, participants completed the pre‐education survey independently on a computer, received the standard SCT education, and then completed the post‐education survey independently on a computer immediately following their session. The pre‐education survey included a demographic survey, a health literacy assessment, and the SCT Knowledge Assessment (SCTKA) (Table 1). The post‐education survey included the SCTKA and asked caregivers to report their satisfaction and feedback about their education session, their intentions to inform their child and family about SCT, and if they were reassured by the education they received. The educator was present while the caregivers completed the pre‐ and post‐surveys but did not answer questions about the surveys and did not review the caregivers’ survey results before providing the education. Participants were also asked to provide their contact information if they were willing to be contacted at a later date to complete a follow‐up survey.

**Table 1:** Sickle cell trait knowledge assessment and percentage of caregivers that answered correctly

SCTKA question and answer choices	Pre‐education (%)	Post‐education (%)	Follow‐up (%)
1. Can a child with sickle cell trait ever develop sickle cell disease? Yes No I don't know^a^	46	84.1	73.3
2. Do both parents have to have sickle cell trait for a baby to be born with SS sickle cell disease?^b^ Yes No I don't know^a^	60.2	76.1	70
3. If one parent has sickle cell trait and one parent has hemoglobin C trait, could they have a baby with sickle cell disease? Yes No I don't know^a^	18.6	88.5	56.7
4. If you have sickle cell trait, could your brother or sister also have sickle cell trait? Yes No I don't know^a^	61.1	80.5	66.7
5. Can you choose which genes are passed onto your children? Yes No I don't know^a^	92.9	92	93.3
6. Can you “catch” sickle cell disease like a cold? Yes No I don't know^a^	86.7	96.4	100
7. Can sickle cell disease cause death? Yes No I don't know^a^	71.7	97.3	93.3
8. What test can be used to find out if you have a hemoglobin disorder? Urine Test Electrophoresis and Complete Blood Count Platelet count Lipid Panel I don't know^a^	44.2	92	73.3

SCTKA, sickle cell trait knowledge assessment.

a “I don't know” was considered an incorrect response.

b Sickle cell trait education at Nationwide Children's Hospital focuses on the reproductive risks of two individuals with sickle cell trait having a child with sickle cell disease.

### Measures

#### Demographic survey

Caregivers self‐reported their gender, age, relationship with child, household income, education level, personal SCT status, and if they knew someone with SCD. Household size was not collected but household incomes ≤$20,000 per year were defined as low because this income level met the Medicaid eligibility criteria threshold in Ohio in 2016, assuming that each household included at least one child with SCT and one caregiver (Ohio Department of Medicaid, 2016). Participants who did not graduate from high school were considered to have low educational attainment.

#### Health literacy assessment

Caregivers’ health literacy was assessed using the validated Newest Vital Sign (NVS) measure (Weiss et al. 2005). NVS scores range from 0 to 6 and low scores suggest a higher risk for low health literacy. Participants with NVS scores <4 were considered to have limited health literacy.

#### SCT knowledge assessment

This study used the SCTKA (Table 1) to quickly assess caregivers’ SCT knowledge. The SCTKA was not validated, but seven of the SCTKA questions were previously published in a study that aimed to determine the impact of newborn screening and genetic counseling on families with children with abnormal hemoglobin traits (Kladny et al. 2011). The SCTKA was developed to be understandable by those with low reading levels, and before enrollment, we pilot tested it on 10 caregivers of children with SCT to ensure readability. While the SCKnowIQ is a survey that has undergone mixed method evaluation and includes items on the genetic transmission of SCT, it was not used in this study because it also includes questions about reproductive health and behavior that are not covered in the SCT education at NCH and takes approximately 30 min to complete (Gallo et al. 2014). In contrast, the SCTKA is eight questions and all of the items on the SCTKA are included in the standard in‐person education. We defined participants with SCTKA scores ≥75% correct as having high knowledge because this was the minimum score the NCH SCT team agreed that caregivers needed to achieve SCT awareness and prior standards for classifying caregivers based on their SCTKA score did not exist.

#### Follow‐up assessment

Participants were sent one email and called up to two times by telephone to complete a follow‐up assessment, if they agreed to be re‐contacted at their initial visit. A separate research coordinator completed these follow‐up assessments with participants beginning 6 months after the final participant completed SCT education. Follow‐up assessments were completed verbally over the telephone; so, participants had to rely on their sustained knowledge to answer the SCTKA questions in real time and could provide feedback about SCT education to someone other than the person who provided the education. Finally, caregivers were asked to report their preferred learning style(s) (verbal, visual, other) and whether they have any learning barriers (visual, hearing, other). A $10 gift card was provided to each caregiver who completed the follow‐up assessment.

### Statistical analyses

Due to nonnormality, nonparametric statistical tests were used. The Wilcoxon Signed‐Rank test was used to compare caregivers’ pre‐ and post‐education SCTKA scores. Pilot testing the SCTKA on 10 caregivers provided point estimates for the sample size calculations. Mann–Whitney U tests and Chi‐square or Fisher's Exact tests were used to compare participants’ demographic characteristics, NVS scores, and pre‐SCTKA scores between those with high and low post‐education SCTKA scores. Nonparametric tests were also used to compare the baseline characteristics of those caregivers who completed and did not complete the follow‐up assessment. Friedman's test with adjustments for multiple comparisons was used to assess changes in SCTKA scores for those who repeated this evaluation at all three study time points. Statistical analyses were performed using the base R statistical package (R Foundation for Statistical Computing, Vienna, Austria).

## Results

During the study period, 374 infants with hemoglobin S‐trait had at least one caregiver attend a SCT education appointment and each of their adult caregivers who presented was consecutively approached to participate. Of these caregivers, 124 of were eligible, 114 enrolled on the study, 113 completed the pre‐ and post‐education assessments, and 30 completed the follow‐up assessment. The most common reasons for excluding caregivers were self‐report of primary language other than English and previously attending an education session at NCH for a child with an abnormal hemoglobinopathy trait. Seventy (61.9%) of the enrolled caregivers attended the education by themselves, 32 (28.3%) attended with another caregiver who enrolled on the study, and 11 (9.7%) attended with another caregiver who was not eligible or chose not to participate. Participants were from a variety of demographic backgrounds, but with the exception of household income, the demographics of the caregivers who completed the follow‐up surveys did not significantly differ from those who only completed the pre‐ and post‐education assessments (Table 2).

**Table 2:** Caregivers’ self‐reported demographic data

	Total cohort *n* = 113 (%)	Caregivers with follow‐up data *n* = 30 (%)	Caregivers without follow‐up data *n* = 83 (%)	*P* value^a^
Age (years)				
18–24	46.9	53.3	44.6	0.810
25–39	48.7	43.3	50.6	
≥40	4.4	3.3	4.8	
Female	77	86.7	73.5	0.224
Relationship with child				
High‐risk caregiver	95.6	96.7	95.2	0.999
Other	4.4	3.3	4.8	
Household income ≤$20,000	51.3	70	44.6	**0.03**
Type of health insurance				
Public	62.8	76.7	57.8	0.202
Private	29.2	16.7	33.7	
Did not report	8	6.7	8.4	
Did not graduate from high school	10.6	16.7	8.4	0.297
Limited health literacy	52.2	56.7	50.6	0.721
Caregivers who know someone with sickle cell disease	45.1	50	43.4	0.681
High‐risk caregivers who report that they have sickle cell trait	37	46.7	31.3	0.199

a *P* values compare demographics between caregivers who completed the follow‐up survey to those who did not complete the follow‐up survey.

Bold value represents statistical significance.

### Pre‐ and post‐education SCT knowledge

Only 38.1% of caregivers had high SCT knowledge at baseline but most caregivers (90.3%) achieved high knowledge immediately after education. Caregivers’ median pre‐SCTKA scores improved from 62.5% to 87.5% after education (*P* < 0.0001). Caregivers (*n* = 11, 9.7%) who did not achieve high SCT knowledge after education had significantly lower median NVS scores (1 vs. 3.5, *P* = 0.029) and baseline SCTKA scores (50% vs. 62.5%, *P* = 0.003) than caregivers who achieved high SCT knowledge, and these two groups did not significantly differ in age (*P* = 0.527), educational attainment (*P* = 0.999), or income level (*P* = 0.754). Table 1 shows the percentage of caregivers that answered each SCTKA question correctly at each time point.

### Sustained SCT knowledge

**Figure 1:**
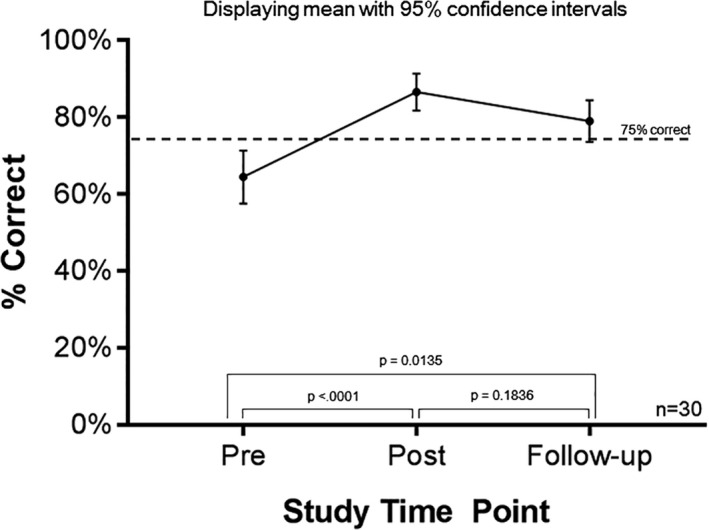
Sickle cell trait knowledge in caregivers who completed pre‐, post‐, and follow‐up assessments.

Caregivers (*n* = 30) who completed the follow‐up assessment had median SCTKA scores of 62.5% at baseline, 87.5% after education, and 87.5% on follow‐up testing. Three caregivers’ SCTKA scores improved between their post‐education and follow‐up assessments, and overall, caregivers’ SCTKA scores continued to be significantly higher than their baseline scores (Figure 1). Over this short follow‐up period, however, half (*n* = 15) of the caregivers had some decline in their SCTKA score and the number of caregivers that scored <75% correct increased from 3 (10%) to 8 (26.7%).

### Intentions to inform and to personally obtain testing

Immediately after education, most caregivers (98.2%) reported they were reassured by the education they received and intended to inform their child of their SCT status (99.1%). Most (92%) reported they planned to tell other family members about their child's SCT status, and on follow‐up surveys, 28 of the 30 caregivers reported that they had done this. We also found that many (60.2%) caregivers intended to personally obtain SCT testing immediately after receiving in‐person education, including 63 (58%) of the high‐risk caregivers in the study.

### Participant satisfaction and feedback

All of the feedback that caregivers provided immediately after receiving the education was positive and none of the caregivers provided suggestions on how to improve the education. Caregivers rated the education easy to understand (94.7%) and stated their questions were answered (99.1%). On follow‐up surveys, the most common way caregivers reported that they learned best was visually (*n* = 18), and three caregivers reported that they had at least one learning barrier (e.g., hearing [*n* = 2], visual [*n* = 1], reading [*n* = 1], or language [*n* = 1]). In contrast, one‐third (*n* = 10) of caregivers who completed the follow‐up testing provided critical feedback about their education session. This feedback suggested using more visual aids, having more interaction and less lecturing, and offering additional follow‐up sessions. Finally, most (*n* = 7) of the caregivers who had low SCT knowledge on follow‐up provided no or only positive feedback.

## Discussion

We found that caregivers’ baseline knowledge is low, but improves with in‐person education. Despite overall caregiver satisfaction with in‐person education, approximately 10% of caregivers did not achieve high SCT knowledge immediately after education and those caregivers had lower baseline SCT knowledge and health literacy. Our follow‐up surveys suggest that while caregivers’ knowledge remains higher than baseline, similar to studies of caregivers of children with SCD (Asnani et al. 2016), SCT knowledge may slowly decline with time. This decline could be partially related to differences in how the SCTKA was administered at the different study time points (independently on a computer vs. over the telephone with a research coordinator), but it is concerning that after only a few months, one‐fourth of caregivers who had received in‐person education had low SCT knowledge.

Our study identifies several potential changes that may improve the in‐person SCT education and future prospective studies are needed to determine if these changes result in higher and more sustained knowledge. More than 50% of caregivers had low health literacy and it was associated with low SCT knowledge achievement. To overcome health literacy as a potential learning barrier, additional education sessions may be needed. The education may also need to include more visual aids and strategies that are effective when educating populations with low health literacy, such as the teach‐back method (Kim and Lee 2016). The content and amount of time spent on some of the topics during the education may also need to be modified. For example, many caregivers at baseline and at follow‐up answered the Hemoglobin C‐trait question incorrectly. This suggests that most caregivers have low baseline knowledge about Hemoglobin C‐trait and that the education provided about this topic may not lead to sustained knowledge. Finally, including caregivers who did not achieve high SCT knowledge in the development and testing of an adapted in‐person education curriculum may also be important, since most of these caregivers did not provide any feedback and additional information is needed to address any of their unidentified learning barriers.

While knowing and understanding the implications of SCT can result in informed reproductive decision‐making, it is important to note that interventions aimed to assist individuals with SCT in their reproductive decision‐making have not resulted in significant intention or behavior changes (Gallo et al. 2016). However, our results suggest that SCT education may provide additional value beyond the potential to inform or change reproductive behaviors. For example, after education, most caregivers reported that they were reassured, intended to inform their child about SCT, and that they had informed other family members about the SCT in their child. Also, many caregivers reported that they intended to personally obtain SCT testing given their own risk of having SCT. This is significant because it supports that education may increase communication about SCT within affected families (Bernhardt et al. 2000), increase the number of high‐risk individuals that definitively know their SCT status, and reduce anxiety.

This study has a few limitations. It was a small, single‐institution study and caregivers who did not present for in‐person education could not be approached. Also, caregivers who reported that their primary language was not English, including some African immigrants who we suspect may still be proficient in English since NCH serves a relatively large population of these families, were excluded. It is unknown if excluded caregivers would achieve similar SCT knowledge scores with the standard in‐person education and this limits the generalizability of our findings. Future studies will need to confirm our findings and address these unique caregiver populations who also need effective SCT education. Second, multiple caregivers of the same infant were allowed to participate and may have resulted in these caregivers having nonindependent survey responses. Third, while more than half of the cohort was classified as low income, assuming only two‐person households may have underestimated the number of low income caregivers in our study and influenced the subsequent income analyses. Fourth, the characteristics of the caregivers that completed the follow‐up assessments was similar to the entire cohort with the exception of respondents having lower household income, but only a small number of caregivers completed the follow‐up assessment because most did not provide contact information that was valid at the time of the follow‐up or did not respond when attempts were made to contact them to complete the follow‐up surveys. Future studies that assess caregivers’ sustained SCT will likely need to include additional strategies to limit participant attrition, such as providing additional incentives to caregivers for completing all of the assessments, confirming and updating caregivers’ contact information more frequently, and scheduling caregivers to complete the SCT follow‐up assessments during their initial visit. Fifth, caregivers’ reported intentions to inform their child of their SCT status may not actually reflect their future actions. Finally, we acknowledge that other education strategies may be more effective at increasing and sustaining caregivers’ SCT knowledge than in‐person education. However, review of other health education intervention studies suggest that it is critical that health education, especially in individuals with low health literacy, include verbal communication (Kim and Lee 2016). Since many caregivers of children with SCT had low health literacy, we suspect that less intensive education strategies that are utilized by some states (e.g., providing a pamphlet in the mail) may not be sufficient to achieve SCT awareness in these caregivers.

In summary, we found that caregivers’ baseline knowledge about SCT is low and improves with in‐person education in most caregivers. Knowledge achievement may be influenced by health literacy and baseline knowledge and may not be sustained over time. This study may inform future prospective intervention studies that aim to achieve high and sustained SCT knowledge among caregivers of children with SCT.

## Conflict of Interest

The authors have no conflicts of interest to disclose.
